# Rituximab Therapy Reduces Organ-Specific T Cell Responses and Ameliorates Experimental Autoimmune Encephalomyelitis

**DOI:** 10.1371/journal.pone.0017103

**Published:** 2011-02-16

**Authors:** Nancy L. Monson, Petra Cravens, Rehana Hussain, Christopher T. Harp, Matthew Cummings, Maria de Pilar Martin, Li-Hong Ben, Julie Do, Jeri-Anne Lyons, Amy Lovette-Racke, Anne H. Cross, Michael K. Racke, Olaf Stüve, Mark Shlomchik, Todd N. Eagar

**Affiliations:** 1 Department of Neurology, University of Texas Southwestern Medical Center, Dallas, Texas, United States of America; 2 Department of Immunology, University of Texas Southwestern Medical Center, Dallas, Texas, United States of America; 3 Health Sciences, University of Wisconsin-Milwaukee, Milwaukee, Wisconsin, United States of America; 4 Department of Molecular Virology, Immunology, and Medical Genetics, The Ohio State University, Columbus, Ohio, United States of America; 5 Department of Neurology, Washington University, St. Louis, Missouri, United States of America; 6 Department of Neurology, The Ohio State University, Columbus, Ohio, United States of America; 7 Neurology Section, VA North Texas Health Care System, Medical Service, Dallas, Texas, United States of America; 8 Laboratory Medicine and Immunobiology, Yale University, New Haven, Connecticut, United States of America; University Paris Sud, France

## Abstract

Recent clinical trials have established B cell depletion by the anti-CD20 chimeric antibody Rituximab as a beneficial therapy for patients with relapsing-remitting multiple sclerosis (MS). The impact of Rituximab on T cell responses remains largely unexplored. In the experimental autoimmune encephalomyelitis (EAE) model of MS in mice that express human CD20, Rituximab administration rapidly depleted peripheral B cells and strongly reduced EAE severity. B cell depletion was also associated with diminished Delayed Type Hypersensitivity (DTH) and a reduction in T cell proliferation and IL-17 production during recall immune response experiments. While Rituximab is not considered a broad immunosuppressant, our results indicate a role for B cells as a therapeutic cellular target in regulating encephalitogenic T cell responses in specific tissues.

## Introduction

Multiple Sclerosis (MS) is an immune-mediated demyelinating disease of the central nervous system (CNS) and an important cause of disability in young adults [1]. The involvement of the immune system in MS is supported by the effectiveness of current anti-inflammatory therapies to reduce clinical symptoms and slow progression of disability [1], [2]. One potential therapy is Rituximab (®Rituxan), which is a humanized mouse monoclonal antibody (mAb) against the human CD20 surface molecule expressed by B cells [3]. Initial case reports indicate that Rituximab therapy was beneficial for MS patients [4]. A phase II double-blind placebo-controlled trial showed a significant reduction of gadolinium enhancing lesions at 4 weeks post-therapy and relapses at 12 weeks post-Rituximab therapy that were maintained for the 48 week duration of the trial [5]. The mechanisms through which Rituximab exerts its effects remain incompletely understood.

This study was designed to investigate the impact of B cell depletion on the T cell response during EAE. It is based on a transgenic mouse that expresses human CD20 (hCD20) under its own hCD20 promoter [6]. Using the MOG_1–125_-induced model of experimental autoimmune encephalomyelitis (EAE), we demonstrate that EAE severity is dramatically reduced in hCD20Tg mice treated with Rituximab prior to immunization or at the onset of clinical signs. Rituximab depletes B cells in the peripheral blood, secondary lymphoid organs and CNS. The absence of disease progression was associated with changes in the CNS-associated CD4 T cell compartment including a decline in MOG-specific T cell proliferative responses and a specific decrease in IL-17 production.

## Materials and Methods

### Mice

hCD20Tg mice were described previously [6]. The hCD20Tg and littermate control mice were backcrossed to the C57.BL/6 (B6) genetic background for >12 generations for these studies. Animal protocols were approved by the Institutional Animal Care and Research Advisory Committee.

### EAE induction and DTH responses

EAE was induced by subcutaneous immunization with 200 µg of recombinant human MOG_1–125_ emulsified in complete Freund's adjuvant (CFA) containing 5 mg/ml of mycobacteria (Difco). On days 0 and 2, each mouse received 200 ng pertussis toxin (Toxin Technologies). EAE severity was scored following a 5-point scale as previously described [7], [8]. DTH responses were elicited by injection of MOG_1–125_ in the ear pinna and net swelling determined at 24 hours as previously described [7].

### Antibodies and recombinant proteins

Rituximab (Genentech) was administered at 100 µg/mouse daily for 3 days. Fluorophore-conjugated antibodies against murine CD4, CD8, CD19, CD45, CD45R and TCRβ or human CD20 were acquired from BD Biosciences or eBioscience. MOG_1–125_ was generated as previously described [9]. MHC class II tetramers containing hCLIP_103–117_ or MOG_38–48_ were obtained from the NIH tetramer core facility.

### Cell culture

T cell proliferation was determined by CFSE dilution after 6 days of in vitro stimulation. IFNγ and IL-17 levels were determined from 48-hour culture supernatants by ELISA (eBioscience).

### Flow cytometry

Single cell suspensions of spleen and CNS tissues were acquired by mechanical disruption through 70 µM mesh. CNS samples were further centrifuged through a 70∶30 discontinuous percoll gradient. Nonspecific binding was blocked with Fc receptor blocking agents and stained with fluorophore conjugated mAbs as previously described [10].

### Statistical Analyses

Where indicated, statistical comparisons were performed using GraphPad Prism5 software. Correlations between continuous and categorical variables were assessed using the Mann-Whitney U test. The means of two normally distributed samples were compared by Student t-test. All other statistical comparisons between groups were examined using one-way multiple range ANOVA test for multiple comparison. P-values <0.05 were considered significant.

## Results

### EAE severity is similar in hCD20Tg and WTLM mice

**Figure 1:**
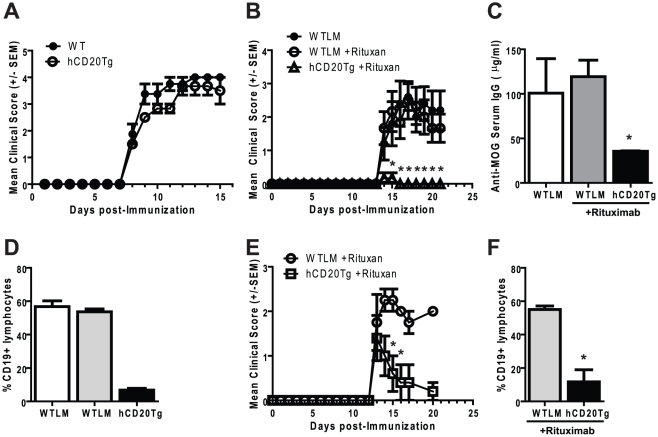
Disruption of EAE pathogenesis by B cell depletion. **Panel A**. EAE severity is similar in WTLM and hCD20Tg mice. EAE onset and severity were monitored using a 5-point scale on WTLM and hCD20Tg mice immunized with MOG_1–125_. These results are representative of at least two independent experiments. **Panels B–D**. Rituximab administration prevents the induction of EAE. WTLM or hCD20Tg mice were either left untreated or were injected with 100 µg Rituximab daily for three days (Day -3,-2,-1). On Day 0, EAE was induced by immunization with MOG_1–125_. **Panel B**. Disease course of WTLM and hCD20Tg mice, EAE onset and severity was monitored using a 5-point scale. Shown are the mean clinical score +/− SEM. **Panel C**. B cell depletion results in reduced levels of anti-MOG IgG in the serum. Serum was harvested on day 21 post-immunization and MOG-specific IgG levels were determined by ELISA. Results shown are the mean IgG concentration +/− SEM. Asterix indicates significant decrease as compared to Rituximab-treated WTLM mice. **Panel D**. Rituximab administration results in rapid depletion of B cells in the peripheral blood. Blood was taken from WTLM or hCD20Tg mice 3 days following the final dose of Rituximab (day 2 post-immunization). B cells were identified by flow cytometry using gates to identify lymphocytes and CD19 expressing cells. Results shown are the mean percentages of CD19+ B cells +/−SEM (*, p<0.01). **Panels E/F**. Treatment with Rituximab reduces EAE severity. EAE was initiated in WTLM and hCD20Tg mice on Day 0. Upon the appearance of clinical signs of EAE, Rituximab (100 µg) was administered daily for three treatments. **Panel E**. Disease course of WTLM and hCD20Tg mice, EAE onset and severity was monitored using a 5-point scale. Shown are the mean clinical score +/− SEM. **Panel F**. B cell depletion in peripheral blood on day 20. Significant differences were determined using an unpaired t-test (*, p<0.05; **, p<0.01). These results are representative of at least two independent experiments with Rituximab and two experiments using the 1F5 anti-human CD20 mAb (data not shown).

The frequency of T and B cells in mice expressing the hCD20 transgene (hCD20Tg) was found to be similar to littermate controls (WTLM), as described previously [6]. In addition, susceptibility to MOG_1–125_ induced EAE was similar in hCD20Tg and WTLM mice (Figure 1A). The onset of disease was found to be on day 8 post-immunization with peak disease on day 12 for both groups. The WTLM and hCD20Tg mice could not be distinguished statistically in terms of peak severity (WTLM 4.0 vs hCD20Tg 3.7) or mean cumulative disease score (WTLM 16.1 vs. hCD20Tg 13.5). Thus, the expression of human CD20 by B cells does not significantly alter the immune response leading to EAE.

### Rituximab treatment alters EAE severity

To understand the impact of B cell depletion on EAE induction, hCD20Tg and WTLM mice were treated with Rituximab for 3 days prior to induction of EAE using recombinant human MOG_1–125_ as described in materials and methods. One group of untreated WTLM is included as a control. Moderate to severe EAE was observed in both untreated and Rituximab-treated WTLM mice (Figure 1B), while hCD20Tg mice were protected from developing EAE. Rituximab-treated hCD20Tg mice showed a significant reduction in serum anti-MOG IgG titers (p<0.02) (Figure 1C), and frequency of peripheral B cells (p<0.01) (Figure 1D). B cells, CD4 and CD8 T cell numbers were reduced in the spleen of hCD20Tg mice even three weeks after the last treatment with Rituximab (Table 1). B cells and CD4 T cells were also reduced in the CNS of hCD20Tg mice 3 weeks after the last treatment with Rituximab. In contrast, CD8+ T cell frequencies were increased in the spleens and CNS three weeks after the last treatment with Rituximab. Minor variance between groups was observed with regard to B and T cell frequencies in WTLM mice either treated or untreated with Rituximab (data not shown).

**Table 1:** B and T cell frequency in prevention model of Rituximab administration.

	Splenocytes		CNS	
	WTLM	hCD20Tg	WTLM	hCD20Tg
**Total cells**	1.21×10^8^	7.60×10^7^(37.2%)	1.50×10^5^	2.00×10^5^(−33.3%)
**B cells**	3.21×10^7^	1.18×10^7^(63.2%)	2.20×10^3^	1.91×10^3^(13.2%)
**CD4+ T cells**	2.13×10^7^	1.44×10^7^(32.4%)	3.54×10^4^	1.05×10^4^(70.34%)
**CD8+ T cells**	1.02×10^7^	7.12×10^6^(30.2%)	1.41×10^4^	3.92×10^4^(−178.0%)

Cells from each organ were pooled from at least three mice per group and counted. Cell counts were then normalized by dividing the total counts by the numbers of mice in each group and then multiplied by the percentage of each cell type as identified by flow cytometry. Total leukocytes were identified by CD45+ events within FSC and SSC gates. B cells were identified using gates for CD19 and B220. T cells were identified by expression of CD3 and either CD4 or CD8. Numbers in parenthesis indicate the percent change in cell counts in the hCD20Tg mice as compared to WTLM controls.

Next, the effects of B cell depletion on active disease was examined by treating hCD20Tg and WTLM mice with Rituximab for three days beginning on the day after EAE disease onset. Rituximab treatment rapidly decreased EAE severity in the hCD20Tg, but not WTLM mice (Figure 1E). This short course of treatment resulted in a sharp reduction in B cells within the peripheral blood (Figure 1F), spleen and CNS (Table 2). Rituximab therapy also showed significant effects on T cell numbers in the spleen (Table 2).

**Table 2:** B and T cell frequency in therapeutic model of Rituximab administration.

	Splenocytes		CNS	
	WTLM	hCD20Tg	WTLM	hCD20Tg
**Total cells**	1.15×10^8^	6.28×10^7^(45.4%)	1.67×10^5^	1.23×10^5^(26.3%)
**B cells**	4.72×10^7^	1.05×10^7^(77.8%)	2.08×10^4^	3.22×10^3^(84.5%)
**CD4+ T cells**	2.01×10^7^	1.47×10^7^(27.1%)	2.39×10^4^	1.57×10^4^(34.6%)
**CD8+ T cells**	9.35×10^6^	6.81×10^6^(27.2%)	7.29×10^3^	5.34mt10^3^(26.7%)

Cells from each organ were pooled from at least three mice per group and counted. Cell counts were then normalized by dividing the total counts by the numbers of mice in each group and then multiplied by the percentage of each cell type as identified by flow cytometry. Total leukocytes were identified by CD45+ events within FSC and SSC gates. B cells were identified using gates for CD19 and B220. T cells were identified by expression of CD3 and either CD4 or CD8. Numbers in parenthesis indicate the percent change in cell counts in the hCD20Tg mice as compared to WTLM controls.

### Rituximab treatment reduces inflammatory T cell responses

**Figure 2:**
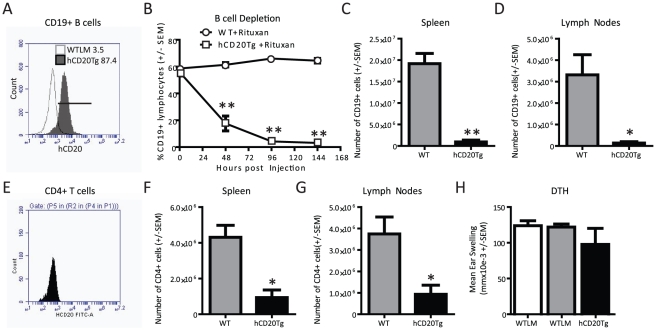
B and T cell dynamics following Rituximab treatment. **Panels A and E**. Expression of human CD20 by hCD20Tg B cells and hCD20Tg T cells. Splenocytes from WTLM and hCD20Tg mice were stained with antibodies to human CD20, CD4 and CD19 and flow cytometry performed. Results shown are gated on CD19+CD4− events to identify B cells or gated on CD19−CD4+ events to identify T cells. **Panels B/C/D/F/G**. WTLM and hCD20Tg mice received three daily injections of Rituximab (100 µg) beginning on day 0. At 144 hours after Rituximab treatment was initiated, tissues were harvested for flow cytometry analysis. **Panel B**. Peripheral B cells are rapidly depleted following Rituximab treatment. **Panel C**. Splenic B cells are depleted following Rituximab treatment. **Panel D**. B cells in the LN (Axilary, Brachial and Inguinal) are depleted following Rituximab treatment. **Panel E**. CD4 T cells do not express human CD20. **Panel F**. Splenic CD4 T cells are reduced following Rituximab treatment. **Panel G**. CD4 T cells in the LN (Axilary, Brachial and Inguinal) are reduced following Rituximab treatment. **Panel H**. Rituximab administration does not prevent priming of inflammatory T-effector cells. WTLM and hCD20Tg mice were treated with Rituximab daily for 3 days (Day -3,-2,-1), followed by immunization with MOG_1–125_ on Day 0. On day 10 post-immunization, DTH responses were elicited by subcutaneous injection of MOG_1–125_ (10 µg) in the ear. The net ear swelling responses were determined at 24 hours. Results shown indicate the mean ear swelling in mmX10E-3 (background subtracted) +/− SEM. Significant differences were detected by unpaired t-test (*, p<0.05; **, p<0.01). These results are representative of at least two independent experiments.

To determine if Rituximab is acting directly on T cells, experiments were performed to examine the expression of hCD20 on B and T cells. As has been described [6], hCD20 was readily detected on splenic (Figure 2A) and LN B cells (data not shown) from hCD20Tg but not WTLM mice. Next, the effects of Rituximab treatment were examined on B cell populations in vivo. B cells were rapidly and significantly depleted in the peripheral blood of hCD20Tg mice following administration of Rituximab (Figure 2B) as well as other anti-human CD20 mAbs including 1F5 (data not shown) [11]–[13]. Three daily doses of Rituximab routinely resulted in rapid (within 96 hours) and near complete depletion of B cells in the peripheral blood. Rituximab treatment also caused a significant, albeit incomplete, depletion of B cells within the SPL (Figure 2C) and LN (Figure 2D) of hCD20Tg mice.

Because our understanding of EAE pathogenesis is largely based on the function of autoreactive T cells, the T cell pool of hCD20Tg mice was also carefully examined. CD4 and CD8 T cell frequencies, as well as activated or memory T cell frequencies in the thymus, SPL and LN were similar in hCD20Tg and WTLM mice (data not shown). The expression of human CD20 was not detected on splenic T cells (Figure 2E), LN (data not shown) or thymus (data not shown) of WTLM or hCD20Tg mice. Splenic (Figure 2F) and lymph node (Figure 2G) CD4 T cells were reduced following Rituximab treatment. The decrease in T cell frequency following Rituximab treatment is likely a consequence of B cell depletion rather than a direct effect of Rituximab on T cells, as previously suggested in Rituximab-treated patients [14], [15].

### Rituximab treatment alters immune responses

**Figure 3:**
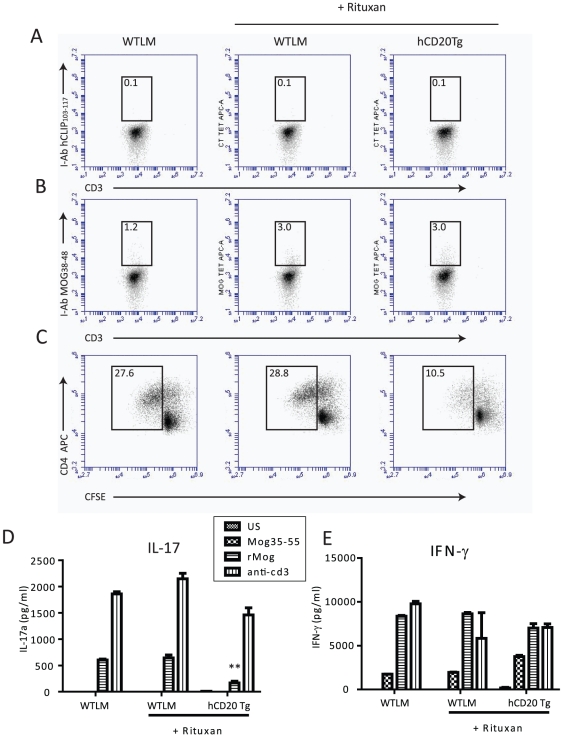
Rituximab administration alters MOG-specific recall responses. **Panels A–E**. WTLM and hCD20Tg mice were treated with Rituximab daily for 3 days (Day -3,-2,-1) followed by immunization with MOG_1–125_ on Day 0. On day 20 post-immunization, bulk draining lymph node cells (LNC) were isolated and recall response determined. **Panels A and B**. Identification of MOG-reactive T cells by tetramer staining. Bulk LNC were cultured for 3 days in the presence of MOG_1–125_ prior to labeling with antibodies to CD3, CD4 and I-Ab tetramers to either human (A) CLIP_103–117_ or (B) MOG_38–48_. Numbers above boxes indicate percentages of T cells in the tetramer positive gate. **Panel C**. Secondary T cell proliferative responses were determined by CFSE dilution assay. LNC were labeled with CFSE and placed in culture with 20 µg/ml MOG_1–125_ and proliferation determined by flow cytometry on day 6 of culture. Results shown are gated on CD4+ events. Numbers indicate the percentage of total cells that diluted CFSE from WTLM and hCD20Tg mice. **Panels D and E**. 48-hour supernatants from the Panel C experiments were examined for the presence of IL-17 (D) or IFNγ (E) by ELISA. Asterices indicate a significant decrease in IL-17 production (p<0.05). Results are representative of at least 2 independent experiments.

In order to better ascertain the effects of Rituximab administration on anti-MOG T cell responses, in vivo and in vitro functional assays were performed. hCD20Tg and WTLM mice were treated with Rituximab prior to EAE induction. MOG-specific delayed type hypersensitivity (DTH) assays were performed 10 days post-EAE induction as an in vivo measurement of peripheral T cell priming potential. Rituximab treatment prior to immunization did not prevent the generation of effective DTH responses to MOG_1–125_ induced EAE (Figure 2H), and MOG-specific T cell numbers were similar in the WTLM and hCD20Tg mice (Figure 3A and 3B). However, MOG-specific T cell recall responses and IL-17 levels were reduced in Rituximab-treated hCD20Tg mice (Figure 3C, 3D). rMOG or anti-CD3-elicited IFNγ levels were not reduced in Rituximab-treated hCD20Tg mice (Figure 3E) or WTLM mice. These results support a role for B cells in promoting MOG-specific Th17 responses in vivo.

## Discussion

Recent results of clinical trials using Rituximab have led to the re-examination of the function of B cells in MS pathogenesis. Here we have described a role for B cells in EAE pathogenesis using the MOG_1–125_-induced model of disease, in which Rituximab administration suppressed EAE severity when given prior to immunization EAE induction or at disease onset.

The effects of Rituximab treatment on EAE were associated with: altered CD4+ T cell distribution in the blood, spleen and lymph nodes; altered T cell recall proliferation; and diminished antigen-elicited production of IL-17. Interestingly, the number of MOG-specific T cells identified by tetramer staining was similar in non-transgenic littermate mice and hCD20Tg mice that were treated with Rituximab at the time of EAE elicitation (Figure 3), although T cell proliferation and IL-17 secretion in response to MOG_1–125_ was decreased in the hCD20Tg mice compared to non-transgenic littermate mice. In fact, Rituximab treatment failed to suppress MOG-specific DTH responses indicating that B cell depletion did not prevent the priming of an autoimmune T cell response. We therefore conclude that the major effects of Rituximab in this model are not due to a direct depletion of MOG-specific T cells during the priming or effector phase but rather point to B cells being critical for the induction or maintenance of neuroinflammation. These results also support the conclusion that Rituximab may function in a tissue dependent manner, and that it does not cause generalized immune suppression [16]. Clinical observations on the organ-specific adverse events associated with Rituximab support our findings [17].

A subset of B cells has been identified in mouse models that can reduce inflammation by producing immunomodulatory cytokines such as IL-10 [18]. Others have further detailed that in peptide-induced EAE models, IL-10 producing B cells could promote remission of EAE [19], [20]. However, our results support a role for B cells in promoting CNS inflammation at the level of maintaining T cell proliferation and Th17 differentiation when EAE is induced with MOG_1–125_. Indeed, a recent publication by Weber et al demonstrates that B cells from MOG_1–125_ induced EAE mice activate encephalitogenic T cells in vitro more effectively than B cells from MOG_35–55_ induced EAE mice [21], and antigen experienced B cells from RRMS patients also induce inflammatory responses by CD4 T cells in a neuro-antigen specific manner [22]. The anti-inflammatory effects of Rituximab may be mediated through the role of B cells as antigen specific APCs to encephalitogenic T cells [22], perhaps through their secretion of IL-6 [23].
